# Capturing the Spatial Relatedness of Long-Distance Caregiving: A Mixed-Methods Approach

**DOI:** 10.3390/ijerph17176406

**Published:** 2020-09-02

**Authors:** Tatjana Fischer, Markus Jobst

**Affiliations:** 1 Institute of Spatial Planning, Environmental Planning and Land Rearrangement, University of Natural Resources and Life Sciences Vienna, Peter-Jordan-Straße 82, 1190 Vienna, Austria; 2 Department for Geodesy and Geoinformation, Research Group Cartography, Vienna University of Technology, Erzherzog-Johann-Platz 1/120-6, 1040 Vienna, Austria; markus@jobstmedia.at

**Keywords:** space–care nexus, single case study, graph theory, spatial semantics, spatial knowledge infrastructure

## Abstract

Long-distance caregiving (LDC) is an issue of growing importance in the context of assessing the future of elder care and the maintenance of health and well-being of both the cared-for persons and the long-distance caregivers. Uncertainty in the international discussion relates to the relevance of spatially related aspects referring to the burdens of the long-distance caregiver and their (longer-term) willingness and ability to provide care for their elderly relatives. This paper is the result of a first attempt to operationalize and comprehensively analyze the spatial relatedness of long-distance caregiving against the background of the international literature by combining a longitudinal single case study of long-distance caregiving person and semantic hierarchies. In the cooperation of spatial sciences and geoinformatics an analysis grid based on a graph-theoretical model was developed. The elaborated conceptual framework should stimulate a more detailed and precise interdisciplinary discussion on the spatial relatedness of long-distance caregiving and, thus, is open for further refinement in order to become a decision-support tool for policy-makers responsible for social and elder care and health promotion. Moreover, it may serve as a starting point for the development of a method for the numerical determination of the long-distance caregivers on different spatial reference scales.

## 1. Introduction

In many regions of the world demographic dynamics and thus unbalanced care support rates lead to new challenges in caregiving for the elderly in rural as well as in urban areas [1]. Particularly, the quality of intergenerational caregiving on the basis of adult children and ageing parents depends on geographical proximity and shifts to the center of the debate on the maintenance of informal domestic caregiving and the appropriateness and necessity for adaption of current (elder) care and health (support) infrastructures, both for the cared-for persons themselves and their caregiving relatives [2,3].

With regard to the latter target group, the so-called long-distance caregivers already receive special attention in the context of the protection of well-being and health promotion by the social and health sciences. From this perspective, the discussion of the quality of life of this target group is considered relevant because from today’s perspective it can be assumed that the trend towards an increasing spatial dislocation of residential locations between the caring adult children and their parent(s) in need of care will continue [3,4]; thus, the number of long-distance caregivers will continue to increase [5,6,7].

In addition, in the Global North there is an ongoing thinning out of (local) intra-family support due to changes in the fertility behavior as well as to changes both in the household structures of families and household structures at old age—the latter mainly due to divorce and widowhood [8].

From the spatial and infrastructure planning perspective, on the other hand, in order to be able to derive conclusions for the future need for ambulant and stationary elder care facilities in different spatial settings, it is of great interest to understand to what extent spatially related aspects—including distances and the associated expenditure of time for commuting for caregiving purposes—may affect the willingness and factual engagement in caregiving.

## 2. Long-Distance Caregiving (LDC) and the Spatial Scatteredness of Life

Long-distance caregivers provide different kinds of support for their older parent(s) in need of care: These range from solely financial support to regular concrete support on-site and thus, relieving the co-resident family members, other local caregiving family members, as well as caregiving professionals, to irregular visits and keeping in (ir)regular touch with the older parent(s) or rather with the professional caregivers via telephone and internet in order to stay informed and involved [2,9].

The kind and intensity of caregiving often is determined by geographical or rather “physical” distance between the places of residence of both the caregiving relative and the older frail person, or rather the effort required to overcome distance [2,6]. However, this can fuel intra-family conflicts, especially between local caregivers and those who have to overcome extensive geographical distances for caregiving reasons [10].

When it comes to health burdens or losses in quality of life, it cannot be clearly answered who suffers more from this challenging situation: Co-residing, local, or (long-)distance caregivers [11].

Nevertheless, it is reasonable to assume that it is of central importance of the elderly or frail parent(s) to live at home for as long as possible, regardless of the spatial-related challenges of caregiving and the length of the caregiving career of the adult child(ren). This in turn particularly strains those (long-)distance caregiving adult children who maintain good relationships with their older parent(s), feel committed to their parent(s)’ well-being, and who are convinced that the cared-for persons are not well enough cared for by professional and informal family support on site.

The debate on long-distance caregiving (LDC) from the social and health care sciences’ perspective is characterized by a tendentious understanding of the challenging living situation of (young) adults who have to overcome long distances for caregiving reasons for persons aged at least 50 during a time period of at least 12 months (National Caregiving Alliance 1997) and “cannot have daily face-to-face contact with the relative” [6].

This target group is defined by means of two spatially related aspects: The “extensive” geographical distance between both the residential locations of the caregivers and the cared-for person [12]—the threshold is set at 50 miles or rather 80 km [10,13]—or the effort of time that is required to overcome this distance. According to Wagner [14] the threshold is at one hour per direction at least. However, there is neither an international consensus on how to define long-distance caregiving [2,7] nor a critical international discourse on when to speak of extensive geographical distances or rather the term “long” [11]. This is astonishing, since the community of long-distance caregivers comprises different sub-groups—amongst others—related to the spatial scale distances and the necessity to cross (inter-)national borders. That may be why an international community of long-distance caregivers has not yet developed. Moreover, the caregivers’ self-perception as long-distance caregivers is only partly related to geographical distance [5].

## 3. Knowledge Gap and Purpose of the Paper

The lack of consideration of further spatially related aspects beyond geographical distances and the effort of commuting in order to explain the particularities of LDC and the related emotional and physical burden and distress—above all in comparison to co-resident and local caregivers—recently has been criticized by Li et al. [11]. They call for a more focused in-depth discussion of the spatial conditions and circumstances of LDC and point out the urgent need for an appropriate theoretical framework for LDC including the role of contextual factors [6] and, so to speak, a spatial turn in the debate of LDC.

Based on the roughness of internationally reported cross-sectional empirical findings, this paper aims to conceptualize the spatial relatedness of LDC from an interdisciplinary spatial research perspective, namely spatial planning and geo-informatics, applying a mixed-methods approach combining single-case evidence with graph theory.

With graph-based structures and semantic ontologies in terms of fundamental spatial data and caregiving information structures, the authors attempt to introduce flexible information modeling, an extensive spatial information structure, and semantic understanding for the creation of a domain open and spatial and infrastructure planning environment. Whereas spatial data standards for data sharing have been implemented within the last decade, the sharing of common spatial semantics for domain specific issues are investigated for standardization within the topics of machine learning and artificial intelligence (AI). Bensmann et al. [15] highlight prerequisites and propose an architecture for geospatial linking, but cannot rely on standardized structures and procedures.

The purpose of the paper is to develop and sharpen the understanding of the spatial relatedness of LDC from a (spatial) planning and geoinformatics perspective, taking into account:

1. How space and spatial aspects impact the engagement in caregiving, well-being, and quality of life of the long-distance caregivers themselves;
2. The significance of objective and subjective aspects of spatial relatedness;
3. In order to model the space–care nexus, considerations on the availability and appropriateness of already existing geospatial data and further requirements;
4. The limits of the conceptual grasp of LDC applying an interdisciplinary mixed-methods approach.

Finally, this paper should be the impetus for starting the discussion on (cross-cutting) methodologies for the quantification of the LDC phenomenon [1].

## 4. The Role of Qualitative and Quantitative Data Sources for a “Single Case” Evaluation

A spatial-oriented discussion of LDC requires a detailed factual analysis with the most detailed data that are available. But data availability is only one specific aspect. Available data need to be interoperable across different thematic domains and qualities. Most spatial analyses intend an automated processing of all information sources and with this support objective planning decisions. Some open information sources already exist under the acquis of Europe’s digital transformation and future-oriented data strategy [16]. But many information sources in the area of health and caregiving are still closed down for the purpose of the general data protection regulation. Enormous effort is needed to complete the required records and create consistent information, like it is done by the European statistical office [17]. Completeness and consistency are just a first dimension that has to be reached. A cross-domain data integration, as it is needed for a comprehensive planning, presupposes semantic interoperability and flexibility for qualitative and quantitative information inputs.

There is an emotional, thematic, and spatial impact on the analysis of the topic of LDC. The spatial dimension relates to the activity centers of the caregivers with focus on connectivity, time consumption, and diversity. Are there more than two activity centers, like work-living and caring, that have to be served?

The thematic dimension concerns the “focus of question”, which may have an impact on the analysis of the LDC topic. In other words, the “focus of question” could highlight the overall situation of the single use evaluation.

The emotional dimension embeds spatial sequences of barriers or encouragement, which can be identified in the evaluation of the specific use case.

Zhang et al. [18] show that the LDC observation, which is done as interview and therefore in form of storytelling, could be distorted by emotional immersion, which is significantly more immersive than spatial immersion. This significance affects perception of time, realism, sense of engagement, sensory cues, emotional aspects, and many more. In terms of attention and image motion, spatial immersion seems to be as immersive as emotional immersion.

A central challenge is the issue of data integration of different sources and especially the mixture of qualitative and quantitative data structures.

According to the recommendations of the United Nations Expert Group (UN-GGIM) [19], the main challenges for the integration of structured data sources are missing keys. In addition, misleading semantics and hardly considered standards make it hard to combine data usefully. Therefore, the United Nations Expert Group (UN-GGIM) recommends following a five-principle model, which starts with the creation of fundamental national geospatial infrastructures, establishment of geocoding mechanisms, and ends with the full interoperability of statistical topics with geospatial reference data.

The main challenge for qualitative data is the creation of structures and semantics that consider space and spatially related emotions. In addition, spatially related emotions are influenced by occurring situations [20], which need to be considered in the data structures, semantics, and even integration mechanisms.

For the example of health situations, long-distance caregivers could play their specific role in epidemiological spreading as it was observed in the COVID-19 pandemic crisis. At least LDC needs to be considered in the actions of pandemic control in order to keep up caring mechanisms.

From the data-driven point of view, the integration of occurring data varieties enhances their importance for the single-use analysis. An evaluation of possible methodologies for describing the common understanding of space, the relation to qualitative information (interview), and the actual state of standardization seems to be appropriate for creating a higher reliance of the results.

## 5. Materials and Methods

### 5.1. Research Approach

An ontological research approach was chosen for the analytical conceptualization and modeling of the spatial relatedness of LDC. For this purpose, conceptual considerations on the spatial relevance of LDC were made from a spatial sciences’ perspective and a suitable approach was sought for the operationalization of spatial relevance through the application of spatial semantics of LDC. Based on this, an analysis grid was developed into which empirical data were fed.

It was decided to test the suitability of the analysis grid using existing primary material from an already existing single case study of a female long-distance caregiver in Austria, who according to Bledsoe et al. [6] represents “the ´typical´ long distance caregiver” [ibid, 305]: Middle aged, highly educated, and with high income.

**Figure 1:**
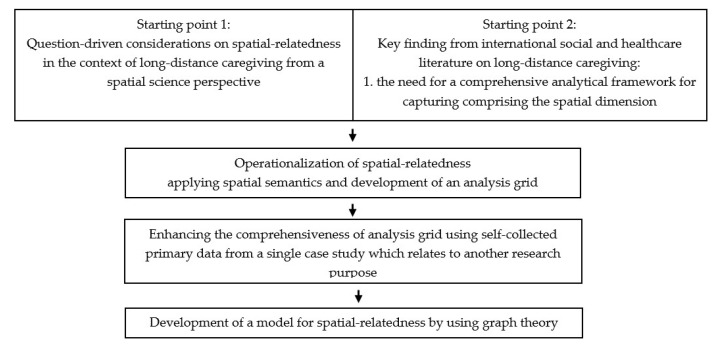
Methodological considerations on the research procedure. Own illustration.

Subsequently, the space–care nexus was modeled using graph theory (see Figure 1).

### 5.2. Operationalization of Spatial Relatedness Applying Spatial Semantics

The conceptualization of the spatial relatedness of LDC requires comprehensive considerations and taking into account not only objective facts but also subjective perceptions and feelings of the people involved in the LDC setting. The latter is done on the basis of the available empirical material from a single case, focusing on the perspective of the long-distance caregiver.

In a first step the term “spatial relatedness“ was operationalized, which means that according to the spatial semantic hierarchy of Kuipers [21], the geospatially related structure has been classified. This spatial semantic hierarchy (ibid.) aims at artificial intelligence with a fully automated spatial decision methodology. From his point of view, spatial metaphors are unique for the communication of spatial relationships and processes because space delivers a preexisting knowledge base [22].

According to Kuipers [21] the information in a spatial semantic hierarchy is split into five layers, which span from a sensory-, control-, causal-, topological- to a metrical-tier. For our approach of applying spatial semantics the causal-, topological-, and metrical tiers are of specific interest.

The metrical tier represents a global 2D geometry in a Euclidian space. In the case of spatial semantics for LDC the metrical tier specifies coordinates, a georeference, and geodetic distances.

The topological tier represents places, paths, connections, and order. It also inherits one dimensional distance. In the case of the LDC spatial semantic the topological tier becomes expressed by the graph model with its nodes and relationships. The properties of the nodes define places and order. The relationships of the nodes define connections.

The causal tier includes actions, causal schemas, and individual views. The causal tier bridges the qualitative interview with topology. Within the spatial semantic model of LDC, the causal tier can be embedded by labels for the nodes and relations. It may even express causal properties if strong positions characterize common valid entities.

In the following the information from the single case was merged with spatial semantics in order to capture the space–care nexus taking into account the considerations of van Broese Groenou and De Boer [23]:

(a) The (assumed) correlation between the physical distance between the places of residence and the commuting rhythm or the length of stay of the long-distance commuting family member with the parent(s);

(b) The perceived need on the part of the long-distance caregivers for intensive engagement in the care of the parent(s) depends on the social environment and the infrastructural quality of the direct living environment of the cared-for old(er) persons;

The idea now was to describe “places” identified as relevant in the context of LDC as well as the relationships between places using spatial semantics in order to be able to provide answers to the following key questions:

- “How far do you have to drive?”/“How far is one willing to travel as a caring relative?”;
- “How difficult is it to overcome geographical distances and how often is a caring relative willing to overcome this distance?”;
- “What are the local living conditions of the person in need of support or rather care that requires (more) engagement on the part of the adult child(ren) living far away from the parent(s) in need of support and care?”

For this reason, building on the relationships between space, concern, and factual engagement in taking over caregiving tasks described in international literature (see Table 1), the spatial-related aspects in the context of LDC considered as relevant were assigned to the semantic categories as follows:

- Geographic distances in Euclidean space (= distances in miles or rather kilometers) (= metric semantics);
- Spatial-related aspects addressing all aspects of overcoming geographic distances and explain (partly) the efforts in the contexts of LDC such as topography, reachability, transport modes (= topological semantics);
- Spatial-related aspects related to the availability of infrastructure supply structures, above all professional and informal care and social support structures in the immediate vicinity of the place of residence of the cared-for persons, which are decisive for a person becoming an LDC (= causal semantics).

### 5.3. Decision on a Specific Single Case, Data Collection, and Particularities of the Empirical Material

The lack of a comprehensive understanding of the spatial relatedness of LDC and the lack of sound and validated empirical evidence justifies the application of a single case study approach [32,33].

The primary data on the single case which in the following builds the empirical basis for the analytical framework for the spatial relatedness of LDC originates from a finding on an Austrian female long-distance caregiving daughter. In order to understand the complexity of the space–care nexus and the relevance of spatial scatteredness of life in the context of LDC over time, the long-distance caregiver was consulted twice: In 2015 and in 2018.

The first consultation took place in May 2015 and was part of a series of face-to-face interviews with five Austrian long-distance caregivers. The survey aimed to draw attention to the spatially related challenges of long-distance caregivers and the relevance of the topic of LDC in connection with social and health planning in Austria for the very first time. The motivation for carrying out this survey was exclusively based on the interest in knowledge and, thus, investigative [34]. In this context, the term “multilocal caregivers” was introduced, which aptly describes the “spatial scatteredness of life” [35].

The explorative face-to-face interviews took place at various meeting points (café, at the interviewees’ workplace), and were based on guiding questions and addressed the following topics:

- The description of the spatial constellation of the places of residence both of the interviewee and the cared-for older parent(s) and the engagement in domestic care (residential locations of the caregiver and the cared-for person(s), degree of kinship);
- The need for support or rather care of the person being cared for and the care mix (professional and informal care, support by the respondents themselves);
- Challenges related to overcoming geographical distance and the consequences for the nature or rather periodicity of support and the quality of life of the (long-distance) caregivers;
- The impacts of LDC on health and well-being of the interviewees and their coping strategies.

Due to the fact that this research did not count as investigative medical research, no approval from an ethics committee was required. The interviewees were informed about the purpose of the survey and gave their verbal consent to publish the findings in anonymized form.

Access to the interviewees on the one hand was provided by the heads of the Viennese nursing care discussion forums (4 out of the 5 interviewees), on the other hand through snowballing among family and colleagues.

At the time of the survey, the interviewees were aged between 40 and 70 years and looked after at least one of their parents. It turned out that three out of the five interviewees met the criteria for being a long-distance caregiver.

The interviews lasted between one and two hours, and were recorded on tape and then transcribed mutatis mutandis by the interviewer, including direct quotations.

One of the interviews was particularly intensive because the interviewee was—also due to her educational background—very interested in the topic and the relevance of the spatial dimension of LDC. Therefore, and due to her ability to accurately express herself verbally, from the very first moment she tried her best to reflect her personal caregiving situation comprehensively and critically, pointing out the cause and effect of extensive geographical distances on her well-being and the amount of on-site support for the parents as well as the challenges and limitations of overcoming distances for caregiving purposes. Furthermore, she did not shy away from looking into the future and also openly addressed the issue of repression of what may yet come.

The ease of the conversation flow as well as the sympathy between the interview partners encouraged the interviewer to go into depth in terms of content, taking into account all the rules of good conversation and respecting the dignity and intimacy of the interviewees according to the Guidelines for Good Scientific Practice of the Austrian Agency for Research Integrity [36].

Finally, the conversation developed into a mixture of biographical–narrative interview following the research questions and problem-centered interview approaching the space–care nexus. At the end of the interview, the interview partners agreed to keep in contact and to continue to exchange on the topic on occasion.

Two and a half years later, in December 2017—in the meantime, the interviewee had changed her job and moved to another province of Austria—the interviewer again contacted the interviewee by telephone with the request to talk again about the caregiving situation. Knowing details of the new place of residence and the workplace (name of the respective municipality), the researcher was interested in finding out to what extent these changes had an effect on the caregiving situation and the organization of daily life.

Therefore, the focus of the second consultation was on an in-depth investigation of the space–care nexus, with particular attention to and taking into account of eventual changes in the health status of the looked after or rather cared-for parent(s), the care–life balance, and the emotions associated with being on the move.

This time she wished to deal with the questions in writing. In addition, she wanted to take enough time for a sufficiently intensive answering of the questions. For this reason, the respondent was asked to describe her living situation in text form and, if possible, to depict her emotions graphically by using symbols.

A theme-centered questionnaire comprising ten open-ended questions was developed, preceded by an introductory text based on the findings from the face-to-face interview in 2015 (see Table 2).

The questionnaire was sent out at the end of December 2017 via e-mail and the respondent was informed about the purpose of the second survey and the intention to publish the findings sometime and in an anonymized manner according to the General Data Protection Regulation of the European Union. The respondent provided her answers at the end of February 2018 and gave consent.

### 5.4. Using Ontologies from a Technological Viewpoint

From a technological viewpoint all given information has to get connected. A connection can be established when the understanding of the connected nodes is correct. A description of the nodes´ and relations´ semantics is accomplished in an ontology. The standardization for connecting data on the web is driven by the World Wide Web Consortium (W3C) [37]. These standards are a good meeting point for exploring the LDC data diversity and its modeling, because it shows a similar characteristic to the diversity of data on the web.

In the following we describe the importance to link location, thematic information, and narratives. We will introduce the main existing ontologies on the web and give an insight into technologies for a prototypical evaluation.

#### 5.4.1. Why Connecting Technologies and Humanities?

Nowadays data science is driven by the integration of different sources of information. Key issues are the relation to space, because most of information originates anywhere, and time, because information always has time relevance. Moreover, the structures of different sources of information are not consistent or even unstructured, which opens up new research fields in data and geoinformation science. For example, Bhatt and Wallgrün (2014) [38] highlight new perspectives for GIS and its methodologies, which effect, on the one hand, knowledge engineering, semantics, and modeling, and on the other hand, analytical processing. Both aspects are driven by a narrative-centered representational and computational apparatus for next-generation GIS.

From a high-level point of view, a knowledge network that incorporates location, structured information, and narratives seems to deliver the most appropriate fundament to govern essential decisions. This form of data integration is important because location is the main link between society, the economy, and the environment [15,19].

#### 5.4.2. Relevant Ontologies and Their Standardization

The main application field for ontologies is the web, where information needs to be found and different junks of information are linked together. This initial characteristic of the web has been further discussed for the definition of notions like Web 2.0 or Web 3.0 [39], which mainly relate to the semantic web. Automation of information retrieval leads to application fields in machine learning and artificial intelligence, which make use of the semantic annotation of features.

Because of this main application field, one of the most impressive ontologies, besides the web ontology by W3C [40], is schema.org [41]. Its defined vocabulary structures the knowledge of the web and is used in search engines and applications beyond. The vocabulary covers features, actions, as well as relations between features and actions. It can be used with different encodings like RDFa, Microdata, or JSON-LD, and is easily extensible with a well-documented extension model (ibid.).

Other extensive ontologies have been created for the knowledge structuring of Wikidata [42], dbpedia [43], or geonames [44]. All of these examples embed a spatial component, a specific description of the spatial thing, and its relations.

Standardization procedures exist for the formats and syntax that describes an ontology and its vocabulary. The semantic content, the definitions of features, and relations are almost not controlled and standardized. Many knowledge networks that are built for specific analysis with linked data use their own internal definitions. This results in the situation that those results can hardly be combined with other results and therefore do not follow FAIR (Findable, Accessible, Interoperable, Reusable) principles [45].

#### 5.4.3. Overview of Possible Technologies and Their Flexibility

Storing semantic content may challenge data storing and accessing mechanisms. Whereas many applications make use of relational database management systems (RDBMS), newer developments enhance performance with noSQL and graph databases. Exchange interfaces incorporate standards for RDFa or JSON-LD. The storage and accessing structures differ for the mentioned DB models.

The authors of this paper assume that the analysis and evaluation of the different information sources for LDC will improve by the technology of labelled property graphs [46], which allow for attributing features, entities, actions, and relations. The spatial aspect is very much often underestimated and needs to be improved. Several initiatives try to enhance and interpret graph structures for spatial data on the web [47].

### 5.5. Development and Structure of Analysis Grid Using Information Based on Single Case

Several initiatives regarding the modeling of spatial relatedness of LDC based on the perspective of the long-distance caregiving person and graph theory must be preceded by an appropriate preparation of the available relevant spatial information and its assignment to the various semantic categories.

For this purpose, an analysis grid was developed, in which all spatially relevant information extracted from the single case was filled in. In terms of LDC, spatially relevant information includes information on environment, arrangements, interfaces, development, perceived challenges and benefits, and place-associated feelings, and was broken down in categories [48].

The categorization was as following:

- Relevant places on different spatial scales (residential community, neighborhood, the flat/house) and their relevance and function for the life, well-being, and health of the both the long-distance caregiving person and the cared-for person(s);
- Spatial constellations of relevant places, above all the places of residence and work of the long-distance caregiver and the place of residence of the cared-for old(er) parent(s), and relationships/connections between places/spaces (paths)—see also Li et al.’s research agenda regarding [11] the emotions associated with places and paths (overcoming distances);
- Characteristics of the direct living environment of the residential community of the cared-for old parent(s);
- (Eventual) changes in spatial aspects identified as relevant for LDC over a 5-year period (2013–2018) and outlook for the future as an LDC.

In order to maintain anonymity to the respondent, subsequently the place names were anonymized; instead of this, a distance matrix (see Table A3) was created in order to portray the relationships between the different places (= paths) and the causal semantics of the places were supplemented by information from other data sources. Although the authors know that a map will present the physical context of LDC and topographical influence on mobility issues best, this visualization has not been created in view of rural situations, where a village consists of a few households, and therefore ethical research codes could not be assured. Maps are planned for follow-up research documentations beyond the single case study.

In order to vividly illustrate the space–care nexus, direct quotes from the interview as well as the written survey were inserted at appropriate places in the analysis grid.

### 5.6. Explanation of Decision for Graph-Based Analysis Structures and Modeling Spatial Relatedness of Long-Distance Caregiving

As shown with thematic clusters, the analysis of an LDC situation incorporates various domains, which exceed the geospatial methodology. Those different domains characterize the data that can be extracted from observations, measurements, and interviews. In general, these data, whenever translated into relational tables, lead to rigid relational models, which hardly express and adapt to the real situation. In contrast, a graph-based model is flexible enough to be extended with individual observations of an LDC analysis. As a result, the LDC ontology that describes the space–care nexus may shape a common applicable knowledge structure for a better interpretation and planning basis.

A graph-based analysis structure makes use of a graph database, which consists of the underlying storage and the processing engine. The storage component is responsible for storing and managing the graphs in a native manner. The processing engine provides a native graph processing, which results in significant performance advantages of an index-free adjacency [49]. In our approach we use the graph modeling and therefore focus on the storage component of a native graph database.

Whereas in traditional relational databases the data are connected with foreign keys and stored in table collections, in a graph database the relationship is the “first-class” object. This leads to the assembling of simple abstractions with nodes and relationships and enables the creation of sophisticated models that are close to the real-world problem [50,51]. Furthermore, graphs are additive by nature. This means that the adding of new relationships, nodes, labels, or even subgraphs does not influence existing queries and analysis. In case of the LDC exploration, a stepwise extension of the model with new insights is easily done.

Beside the relations and nodes in a graph model, labels are used to categorize nodes in the model. Some of the nodes are persons, others are places, or transit modes. A more detailed description is accomplished with properties, which are arbitrary key–value pairs in the form of simple data types, like strings or numbers. For some graph algorithms it is useful to add properties to the relations as well in order to describe the connections with additional metadata, like the quality, time, or weight. The result then is a labeled property graph [52].

The categorization of nodes and relationships leads to a specific ontology, which is a common valid structure of semantics—in our case for the LDC situation. This semantic structure with its embedded “naming” of nodes, relations, properties, and attributes is domain specific. A comprehensive standardization is missing. Kuhn [53] tries to collect a common understanding for geospatial semantics and differs between the semantics of expressions, semantics of services, semantics of interfaces, and geospatial semantics [47]. From a pragmatic implementation-oriented view, geospatial semantics is different with some important properties [54] that influence the modeling of LDC:

1. Human perception and social agreements directly influence geospatial information, when objective measurements are mixed with subjective judgements. A mapping between both [55] is a main challenge which helps to make geospatial information more meaningful;
2. The identification of geographic entities is used to enable georeferencing and better translation capabilities. These identifiers are used to link entities and, in the case of knowledge graphs, to establish relations;
3. The situation of LDC and its geographical reference is not a static view, but a process in time and space [56]. Distances, directions, and relations move and therefore the overall situation changes. A formal description of the LDC theme needs to consider processes in time and space;
4. The modeling with geographical information incorporates vagueness, uncertainty, and different levels of granularity. The relation of these granularity levels of geographic information [57] with the semantic granularity of ontologies [58] and a qualitative information coming from an interview is an essential part of a valid knowledge graph model for LDC.

These key characteristics of geospatial semantics give reasons for the theory of semantic translation, which is capable to join geospatial information with thematic information across the boundaries of their different communities [59] and data characteristics.

The ability of semantic translation to link up different knowledge communities even leads to new formal methods in qualitative spatio-temporal reasoning, actions in spatio-temporal dynamics, and new perspectives for the development of the foundational spatial informatics. In geospatial narrative semantics we seek to define formal models of spatial and temporal relations that consider aspects of space, topology, direction, distance, size, traversal distance, and so on. The resulting field of qualitative spatio-temporal representation and reasoning (QSTR) has been evolved as specialized discipline within artificial intelligence [38,60] and could assist a formal evaluation of LDC.

The intention of an LDC ontology and a spatial knowledge model is to create a common formal understanding, which is stable enough that it can be used to support strategic planning decisions on elder care. From the authors’ point of view this robustness depends on the identification of evaluation nodes and consistent relations.

## 6. Case Vignette

In the following, necessary (spatial related) information on the single case—comprising the long-distance caregiver and the cared-for parents—is provided in order to give a comprehensive insight into the specific caregiving situation.

### 6.1. Demographics of the Long-Distance Caregiver

- Female, in her 40s, university degree in spatial science;
- Employed: At the time of the first interview working as a teacher, currently employed in career guidance;
- Living alone and childless: *“Structural deficiencies of regional labor markets create personal destinies.”* … *“I unwillingly have no children. I’ve changed jobs often. … I often wonder what it will be like when I’m older.”* That is why she feels like *“the last link in a chain”*;
- Being a long-distance caregiving daughter since 2013, but does not consider herself a caregiver: *“I am an intercessory, supportive, hopefully mentally uplifting daughter who is worried about her parents”*;
- She has a sister and a good relationship with her and exchanges information with her about their parents. *“Yes, the parents are a subject of our talks. The sister is burdened by the job. There are no grandparents. She has tried to return to […]. But there is no suitable job opportunity. She holds a university degree.”*

### 6.2. Character Traits and Attitudes

- Her heart beats for the countryside and she is closely emotionally tied to her parents and her municipality of origin. For this reason, she can imagine returning to Z, the municipality where her parents are still living. *“It’s the roots, the identity. Both compensate for the deficits that I face in the city.”* … *“We [her sister and herself] are attached to the parents’ house.”* Additionally, in her opinion, family cohesion can compensate for the infrastructural deficits in the countryside. At the same time, she mentions that she has no friends in her municipality of origin, because they moved away, too;
- Her central concern: Staying with her parents as often as possible—not only for caregiving reasons, but also because she likes to come home. *“For me distance is not a relief, it is a burden, because I have a close, positive, friendly relationship with my parents.”* She does not feel like a victim; she likes what she does. *“On the part of the parents there is no pressure at all.”*

### 6.3. Employment and Migration Biography

- She was born in Austria and grew up in the countryside *“in a political district with high unemployment”*;
- Since university days she has been living and working in urban areas, having several centers of life;
- In 2013, she moved to X *“for the love of her profession”*, as she could not find an adequate job in financial respects. *“This is a problem for people who are living alone: They earn too much to die, too little to live.”* If she had stayed in […] with a badly paid job, *“it would have all gone up in the car fuel”*;
- She has always maintained regular contact with parents via information and communication technologies: *“The virtual connection helps to make things a little easier. You have the feeling that you are there. But it doesn’t take away the guilty conscience that you still have, because you can’t give immediate support when it counts”*;
- She has found commuting a burden, especially in the past: *“The quality of life goes down the drain. … doing the housework, seeing that food is there and the laundry is done … But the household activities remain the same”*;
- In 2018 she changed her job and returned to her former place of work and education. Since she has been living here again, the time and effort required for LDC has significantly reduced. Now it takes her about an hour by car getting from Y to Z, three hours getting from X to Z: *“That is what makes the difference.”* Returning to Y was accompanied by an increase of the frequency of visits and a shortening of the visit interval: *“Yes, the frequency has increased significantly, because the journey is now only one hour in one direction. This means a maximum of two hours of concentrated car driving on the motorway on weekends.”*

### 6.4. Extra-Familial Social Networks

*“In Y, I built my social networks over the years. In X, I have no social networks. But I wanted to finally pursue my dream job. … I meet my friends who are living in Y every three to four months. This year (as of the time of the first survey in 2015), I met them once for a cup of coffee.”*

### 6.5. Medical History, Living Environment, and Social Network of the Cared-for Parents

- Medical history started at the beginning of 2013: one parent suffered from brain cancer (four surgeries between 2013 and 2017); she moved to Z before the diagnosis;
- One parent suffers from heart disease since 2014 (a surgical procedure was undertaken in the same year);
- Both of the parents are physically impaired, one parent takes medication regularly; both parents are not allowed to perform heavy lifting and should avoid exposure to direct sunlight;
- Especially the time immediately after the first surgical intervention on the mother was difficult for all of them: *“Leaving […] was not easy, leaving the parents behind, who were overstrained. The mother was always a central figure (note: In the household, there was classic division of tasks between the two), the father was in shock … But it was good luck that the sister was on maternity leave at this time.”* (Note: Her sister now (as of 2015) lives in X, the same municipality of residence and work of the interviewees). During this time, the respondent took over work in the parents’ household and in the garden, whenever possible;
- The situation has calmed again: car driving, climbing stairs is again possible for both of them. The living environment itself is no longer a problem (the house is a bungalow): *“That was good luck.”* But gardening is still a problem. Living independently in their own four walls is still possible to a large extent, there is no need for accompanying co-resident professional caring support;
- One parent has to go to hospital regularly for check-ups. The father’s brothers take over the transport service to the therapies in the hospital. *“Taking the bus would have been conceivable, but there is no public transport*”;
- *“The parents’ social network is the family. The father has four brothers, one of whom is a direct neighbor. … The parents’ friends weren’t any help to them, but the father’s family was … There is no additional support from neighbors, associations, or the church.”*

### 6.6. Outlook on the Future as a Long-Distance Caregiving Daughter

- On the part of the parents there is a tendency to want to live closer to the children, but *“it is difficult to resettle someone of this age from a rural region to another place. They are not urban people.”* Moreover, *“the housing situation also ought to be clarified”*;
- When observing the ageing of her parents, the interviewee thinks about what basic infrastructural infrastructure should be available in the parents’ municipality of residence. “*I critically observe the development: Ongoing concentration in the metropolitan areas, the rural regions are lagging behind. … Grocery stores, small retail shops, public transport and buses are rare. Not imaginable, if you live in X.*” With this, the interviewee expresses her concern about the changing structures, above all the thinning out of the infrastructure, which is important to maintain the quality life at a very old age;
- Regarding whether she would talk to the mayor about domestic or stationary care facilities and opportunities, the interviewee replied: “*When I am here, I don’t have time for that.*” Furthermore, she is not in Z during the mayor’s office hours;
- Although she is anxious about the father’s physical fitness [61], she currently has no concrete reason to worry about the distant future. Nevertheless, she has already talked about nursing and care such as the choice for a certain nursing home: “*… unimaginable, because up to now all of the family members have been cared for by relatives and not in a nursing home.*”

## 7. Results and Discussion

### 7.1. Breakdown of Provided Spatially Related Information

This section discusses the meaning of places and the memories and emotions associated with them.

In her life, she has already had three (2013–2017) or rather two focal centers of life (since 2017) with which she associates certain memories and emotions in the context of LDC (see Table A1, Appendix A):

On the one hand, these are the municipalities in which she lives, works, and maintains friendships and social contacts.

X = place of work and residence at time of survey 1 (interview in 2015)
Y = place of work and residence at time of survey 2 (written survey in 2018)
Z = municipality of origin and residential municipality of the cared-for older parents

Referring to these centers of life, the interviewee identifies specific relevant places that were or still are of particular importance to her due to the activities carried out there. Moreover, the interviewee points out the importance of communities as well as other persons and aspects.

At the time of the first consultation in 2015, it seemed to her that her whole life was thoroughly organized: “*I feel like I am living by the clock. No matter where I am, I think about the fact that I will have to leave soon. …The return trips from the parents to X must be well planned. I want to be back in X by 5 p.m. at the latest, otherwise I will not get a parking space. Otherwise I will be forced to make a detour, park the car somewhere else, and continue by public transport. It is a tension and physical challenge.*”

In order to better understand the emotional burdens of commuting for caregiving reasons and emotionally being torn due to the spatial scatteredness of life, the long-distance caregiver was asked to describe her emotions associated with being on the move (see Table 3)

### 7.2. The Graph-Based Knowledge Structure

The single case study gives a very detailed, but also individual relevant image of LDC. It is nearly impossible to relate the individual relevant information to spatial infrastructures, which will allow for a prospective spatial analysis of LDC.

From the interview, we can observe spatial centers of life, their related associations, spatially related characteristics of long-distance caregiving and the dependency on supporting infrastructures and the influence of emotion. All mentioned elements are somehow related and embedded in a story.

According to [62,63] a graph-based semantical structure helps for reasoning and efficiently answering technical questions. In addition the works on “spatialization of narratives” [64] and the “finding of answers with knowledge graphs” [65] indicate that graphs are a promising approach to relate our single case study of LDC to a common spatial understanding. This first step of modeling LDC information has the aim to bridge individual views to common spatial infrastructures and spatial knowledge graphs [66,67,68].

**Figure 2:**
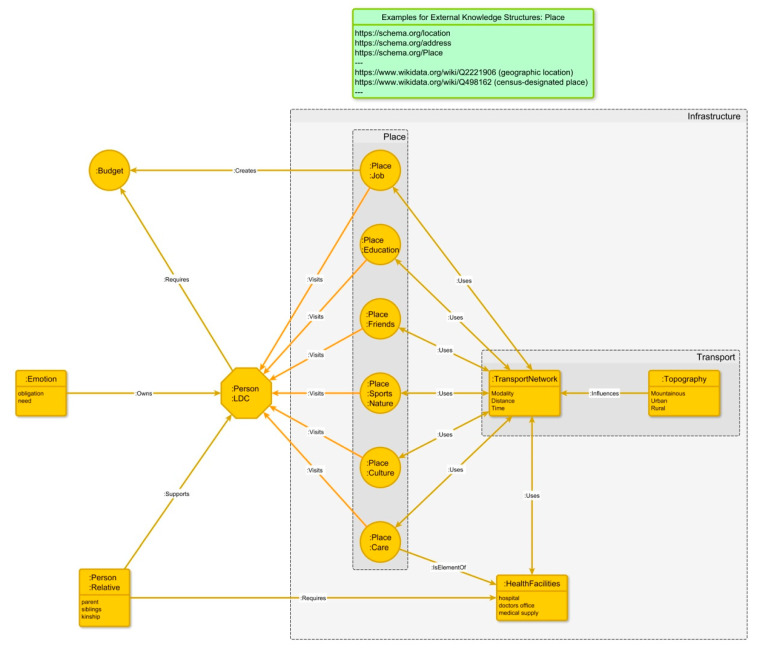
A graph model concept based on long-distance caregiving ontology—including the knowledge graph of a single case study, its most relevant spatial clusters (place, transport, infrastructure), and an exemplary embedding into existing knowledge structures, like schema.org, Wikidata, dbpedia, etc. (on the top of the figure). Own illustration.

For the modeling of LDC information (see Figure 2), the authors of this contribution made use of a basic LDC ontology, which collects information elements and their connections [69,70]. This LDC ontology is an information structure on the basis of the interviews of the single case study and observations of LDC literature. The main findings have been listed earlier in this chapter with the case vignette and spatial centers. Table A2 (see Appendix B) and Table A3 (see Appendix C) show relevant places and their spatial-related attributes from the perspective of the long-distance caregiver as well as a distance matrix. All these elements have been categorized in a way that the connection to “external” existing knowledge graphs becomes enabled and data integration with spatial data infrastructures is supported [71,72].

On the basis of the single case evidence, infrastructure is mainly built up by the variety of places, transport, and health facilities. The variety of places has been expressed by the table of relevant places in Table A2 (see Appendix B). Transport roughly consists of the TransportNetwork with its properties of modality, distance, and time, and topography with a first rough topographic classification to mountainous, rural, and urban. Health facilities cover the properties’ hospital, doctors’ office, or medical supply. The authors would like to express that the classifications are a first step towards a comprehensive LDC ontology on the basis of international literature and the single case study. It needs to be expanded for meaningful applications.

The LDC ontology is a first model which is a topic for further research. We observed that different kinds of places are relevant for LDC. All those places have an impact on the possibilities, willingness, and burden for the LDC.

Although place is defined only once in several existing knowledge graphs [41,42,43], it has different additional meanings for the LDC´s perspective. To a large extent, those meanings can be described and enriched with common points-of-interest (POI) collections, which become increasingly available from authoritative sources. Other POI are of individual character that is expressed by a person´s social environment, interest, and emotions [20].

One element that is often used for the definition and categorization of LDC is the “TransportNetwork”, especially its properties of modality, distance, and time. Modality describes the kind of transport, such as train, car, or airplane. Although the TransportNetwork on one hand is an important element for LDC and a central part of the infrastructure, it is, on the other hand, only a secondary element from the LDC point of view. This means that the simple travel distance and time to the caring person is not a sufficient dimension to categorize an LDC environment. Instead, the variety of places, their persistence, topography, and topological structures need to be considered as it is indicated in the LDC ontology.

Health facilities cover hospitals, doctors’ office, and medical supply. These properties are elements of specific places (with the property “care”) and use the transport network as many health facilities may cause additional traveling. Health facilities from the knowledge network point of view are required for the cared-for person(s) and also cause visits for the caregiving person.

## 8. Conclusions

This paper is the result of a first attempt to comprehensively analyze and operationalize the spatial relatedness of long-distance caregiving by combining spatial sciences’ and geoinformatics’ considerations and developing an interdisciplinary mixed-method approach combining primary data from a single case with graph theory.

From the spatial data science point of view, we can conclude that the LDC ontology is a first step towards integrating information of a single case study into spatial knowledge infrastructures. The matching of individual expressions to common semantic definitions and even to external knowledge graphs opens additional accessible data sources and densifies information, e.g., points of interest, that is needed for a more objective LDC spatial analysis.

We were able to prove the linking to external knowledge graphs works in principle. We observed that neither the specific topic of LDC is covered in currently available knowledge graphs [41,42,43] nor authoritative data sources with appropriate information interfaces, like GeoSPARQL, exist. The last is already a topic of European legislation for 2021 as European Open Data Directive [73].

The extension of knowledge graphs for LDC and the creation of use cases for spatial analysis as well as planning will be further steps for research and a feasibility study. The openly extensible knowledge graph of Wikidata [42] offers an interface and guidance for enhancing the knowledge structure. In the case of local proof of concepts [74,75] the connection to external knowledge graphs and semantic definition registries is an obligation. It enables data integration and a more objective spatial analysis for the topic of long-distance caregiving, which is a focus for future research.

The elaborated conceptual framework could serve as a starting point for a more detailed and precise interdisciplinary discussion on the spatial relatedness of long-distance caregiving and, thus, is open for further refinement in order to become a decision-support tool for policy-makers responsible for social and elder care and health promotion.

Being aware of the methodological and content-related limitations of this approach, we hope that the model will be applied in different countries and on different spatial reference scales and receives validation and extension by as many (longitudinal) empirical findings as possible, in order to:

1. Grasp the principles of the space–care nexus in the context of long-distance caregiving quickly and comprehensively and thus make visible the heterogeneity of the long-distance caregivers in terms of their burdens and needs;
2. Stimulate a critical discussion about geographical proximity, which goes beyond a categorization according to distance classes [11], and the limits of the separability of objective and subjective components in terms of content;
3. Take greater account of the importance of the time dimension of long-distance caregiving careers and the variability of relevant aspects in order to better be able to explore the relevance of the time scale as well as the limits of interpolation between different points in time;
4. Initiate a spatial turn within the debate on the various issues of long-distance caregiving with regard to the discussion about the ability to influence spatial-related aspects in order to maintain well-being and quality of life of all persons involved in long-distance caregiving and, thus, to critically reflect on the suitability or accuracy of strategic demand planning in the context of elder care and health promotion, which, up to now, exclusively is based on demographic indicators and to raise the awareness of the issue of long-distance caregiving among key players in social and health care [Fischer and Jobst 2019];
5. Further develop methodologies for the numerical determination of long-distance caregivers at (pre-defined) spatial scales and in spatial settings (urban, rural, suburban, remote).

For this purpose, it would be valuable to: 1. On the one hand, apply the analysis grid and the graph model to other people involved in long-distance caregiving (e.g., the cared-for persons themselves, co-resident (family) caregivers, local caregiving (family) members, as well as health and social care professionals); 2. on the other hand, evaluate the model by multi-professional teams of researchers—involving representatives of the spatial sciences’ and geoinformatics—and therefore critically examine the suitability of the model as being a part of a decision-support tool in social and health care planning.

## Figures and Tables

**Table 1:** Considering the space–care nexus applying spatial semantics taking the viewpoint of the long-distance caregiver based on (empirical findings in) international literature and own assumptions. Own illustration.

Spatial Semantic Category	Evidence-Based or Assumed (Very Likely) Relevant Spatial Aspects	Reported and (Assumed) Implications for the Long-Distance Caregiver
Metric (position of relevant places and distance related to Euclidian space)	Geographical distances between the places of residence of the caregiver and the cared-for person (national as well as international) [9]	Ambivalence of feelings [24] and “unique difficulties that are less frequently experienced by local family and friends providing care” [25] Feeling of being excluded and under-informed [4] Being expected to keep in contact with the old(er) parent(s) in need of support or rather care [26] Having the feeling of not doing enough for their elder parent(s) [2,10]
Topological (traverses between places/influence on reachability)	Topography Connection of places of residence to high ranking modes of transport (i.e., motorways)	Efforts for overcoming geographic distances [7] Maintaining work/life/care balance [2] Influence on kind of provided support for and visiting the cared-for person [3,6] “Watching the deterioration of their loved one” [7,27] Use of telecommunication to keep in contact with the cared-for person or rather to stay informed [28,29,30] Cost expenditure and choice of means of transport emotional stress [7] and opportunity to take advantage of health promotion offers [1]
Causal (attributes/qualities)	Availability and quality of public transport [31] Availability of informal and formal elder care support and infrastructure in the residential municipality of the domestic cared-for person ((potential) co-resident caregivers, local caregivers, neighbors, friends) Quality of the built environment of the cared-for person (construction-related barriers, maintenance of the garden)	Reasons for being worried Determination of amount of engagement [31] Reason for engagement in on-site domestic care and support for the cared-for person [7]

**Table 2:** Questionnaire for the second consultation in December 2017.

Themes	Introductory Texts and Questions (Verbatim)
Current state of health of the parents and requirements for the respondent as caregiving family member	*“In our first interview on 19 May 2015, you described yourself as a caring daughter who acts as she does, of her own free will and without pressure from her parents.”* Question 1 *“Please describe the course of your parents’ illness since May 2015 and the associated time and—if you wish to do so—the psychological requirements for you as a caring daughter.”*
Spatial centers of life and space-related associations	*“Over a period of xy years (please add) you had three spatial centers of life: X–Y–Z.”* Question 2 *“In retrospect, what do you associate with X?”* Question 3 *“If you had to depict X graphically, which places and emotions would you depict graphically, and which symbols would you use for this?”* Question 4 *“What do you associate with Y?”* Question 5 *“If you had to draw a picture of Y, which places and emotions would you draw, and which symbols would you use?”* Question 6 *“When you think of Z, then you think of …”* Question 7 *“If you had to draw a picture of Z graphically, what places and emotions would you draw, and what symbols would you use to do so?”*
Emotions associated with being on the move	*“In the first interview you addressed the challenge of car driving.”* Question 8 *“What do you feel when you are driving on the motorway* *(a) from Y to Z,* *(b) from Z to Y?”* Question 9 *“What do you feel when you pass the place-name sign of Z* *(a) upon arrival,* *(b) upon returning to Y?”* Question 10 *“Did the return to Y have an influence on the frequency of commuting between the different places of residence? If so, to what extent? If not, why not?”*

Notes: X = place of work and residence at the time of the first interview in 2015, Y = place of work and residence at the time of the second survey in 2017, Z = place of origin and place of residence of the cared-for older parents.

**Table 3:** Feelings of being on the move.

**Feelings on the Road**	
**On the way to the parents (Y → Z)**	**On the way back from the parents (Z → Y)**
*“Joy to see the parents”*	*“Focusing on the social environment”*
*“Inner well-being, coziness, inner brightness”*	*“Friends, colleagues, social network”* *“Reliability, cordiality, communication”*
*“Roots, security”*	*“Job”*
*“Responsibility”*	
*“Focus on important things”*	
**Feelings on Arrival and Departure**	
When she passes the Z’s place-name sign, she feels …	
On arrival at the parents (Y → Z)	On departure from the parents (Z → Y)
*“Inner well-being, happiness and joy”* *“Simply a good feeling to be always welcome”*	*“Looking forward to the next visit, hoping that the parents are well and that they enjoy life in the meantime”* *“I am glad that the distance to Y is only an hour’s car drive and that I can go to Z anytime I need to”*

## Appendix A

Table A1 breaks down the gathered information related to the spatial scatteredness of life as well as place-related associations.

**Table A1:** Spatial scatteredness of life and place related associations.

Places (Activities/Functions)	Communities (Activities/Functions)	Other Persons and Aspects	Associated Memories and Emotions
X (urban municipality)	Family *(“sister and nephew”)*		*“My dream job”* *“New friendships”* *“Inconvenient communication with the parents using information and communication technologies”*
Her first flat (located in the city center)	*“ICT-supported communication with the parents”*	*“The renter”* *“Cultural activities, tourists, pedestrian zone”*	No additional information provided
Her second flat (“the room”)	*“Working”* *“ICT-supported communication with parents”* *“Making new friends with both room colleagues”*		*“Little space”*
The flat of the sister	*“Family, nephew, being godmother, babysitting, talking”*	No additional information provided	No additional information provided
At work	*“Colleagues, interactive working with the pupils, new friendships and leisure activities with colleagues”*	No additional information provided	No additional information provided
Y (urban center)	*“Friends and social network”* *“Job, education”* *“Sports, nature, culture”* *“My home”*		*“Socializing and cordiality”* *“Education, maturing, development”* *“Satisfaction and happiness”*
The flat	No additional information provided	*“The view, nature, living space”*	No additional information provided
University	No additional information provided	No additional information provided	No additional information provided
Z (rural municipality)	*“Parents, grandparents and family”* *“Social contacts”*	No additional information provided	*“Love, childhood, coziness, roots, being earthed, trust and openness, security, reliability, home”* *“Honesty, down-to-earth”* *“Wideness, nature, and silence”* *“Responsibility”*
The parents’ house and garden	No additional information provided	No additional information provided	No additional information provided

## Appendix B

Table A2 gives an overview over relevant places and their spatially related attributes from the perspective of the long-distance caregiver.

**Table A2:** Relevant places and their spatially related attributes from the perspective of the long-distance caregiver (“spatial-related scatteredness of life and ambivalence of feelings”).

	Relevant Aspects Associated with the Dislocation of the Places of Residence, the Spatial Scatteredness of Life, and the Challenge of Overcoming Distances at the Time of the First Consultation in 2015					
Relevant Places	Number of Relevant Places and their Function	Reasons for Staying/Being Present on Site	Emotions	Other Relevant Attributes of the Places	Relevance for LDC Situation and Influence on Well-Being and Quality of Life of Both the Long-Distance Caregiver and the Cared-for Old Parent(s)	Implications for LDC Situation as well as for Well-Being and Quality of Life of Both the Long-Distance Caregiver and the Cared-for Old Parent(s)
Places of residence of the long-distance caregiver	Two places of residence: Place of residence 1 (X) = municipality of work = secondary residence = place of residence of the sister Place of residence 2 (Y) = place of education = primary residence = place of residence of friends Third place of relevance (Z) = municipality of origin = place of residence of the parents	Dream job; purpose-oriented stay; staying as long as necessary rare staying; visits as often as possible every weekend (Friday afternoon to Sunday afternoon), if possible, and during school holidays	Emotional ties (sister and nephew) City Greater emotional bonding in comparison to place of residence 1 (X) Here is where the heart beats.	City, very good job opportunities as well as infrastructure and cultural offers City Rural municipality, an hour’s car drive from the next urban core zone [76]	Spatial scatteredness of life and inner conflict commuting as a burden Neglecting of extra-familial social networks	Reduction of number of places of residence: 2 **→** 1 Reduction of number of spatial centers of life: 3 **→** 2 From now on 2 positively connotated centers of life Shorter distances between the places of residence of the caregiver and the parents imply shorter intervals between the visits
The flats of the long-distance caregiver	At place of residence 1: A base during the week place of work and also cordiality at place of residence 2	Purpose-oriented; daytime marginal times; a place to work No further information	A place of retreat	Co-residence with two other persons rental flat (no further information)	Cost-effectiveness and expediency	
Place of residence of the cared-for parents	= place of origin/place of childhood = place of care and support = place of leisure = a place to rest	Staying as long as possible as well as often as necessary; usually on weekends (Friday afternoon to Sunday afternoon) and during school holidays	Here the interviewee feels at home and secure	Mentioned available infrastructure (as of survey 1): One practitioner, pharmacy, motorway exit, the latter is considered to be *“an advantage for the municipality”* The proximity to nature family support	Observation of infrastructural changes (thinning out) and worries about care needs of the parents in later life (they want to stay there, in their own built house; informal domestic care is a long-standing tradition in the interviewee’s family She is thinking of returning to Z after retirement	
The parents’ house	= place of support = place of cordiality = accomodation during the visits	Whenever she visits her parents or looks after them	Place of childhood Both the interviewee and her sister are emotionally attached to the parents’ house; the interviewee would like to take it over one day	The house and the garden; not barrier-free	She lives there when he’s with her parents. Therefore, no additional distance is to be overcome as well as no additional expenditure for commuting apply stress and bad conscience: *“Can’t support the parents in gardening”*	Prospects for a probably long-lasting caregiving career: The parents want to stay in their own four walls *(“You don’t plant an old tree”)*
The motorway	The direct and fastest connection between the two places of residence (caregiving daughter & parents)	As long as necessary, as quick as possible	The fastest connection; nevertheless, the motorway is experienced as a burden: *“It is necessary to concentrate fully for three hours”*	Dangers: Traffic & construction sites	Distance and topology justify the choice of transport mode (own car) = explanation of physical and psychological strains in the context of overcoming distances (X **→** Z) “*The weekend trips to my parents’ house in Z meant all things considered more than six hours of concentrated driving on the motorway*”	
Health care facilities for medical and nursing care (hospitals, medical practices)	Place of medical treatment for the parents	No relationship identified			She would like to support her parents by taking them to their medical treatments; but due to the extensive distances to overcome she can’t	

## Appendix C

Table A3 shows the functional relationships between the relevant places; distances, time efforts, and emotions in the context of overcoming distances (always on the move) from the perspective of the long-distance caregiver.

**Table A3:** Always on the move: Functional relationships between the relevant places; distances, time efforts, and emotions in the context of overcoming distances from the perspective of the long-distance caregiver.

Routes and Directions	Distances and Time Efforts	Implications for the Life as Long-Distance Caregiver	Changes between the Two Survey Dates and Consequences for the Spatial Behavior of the Long-Distance Caregiver and the Amount of Support for Her Parents
X → Z Z → X	Road distance (in km): 270 Travel time (by car): 2 h 53 min	Explanation of why she can only support the parents selectively and cannot visit them regularly She can hardly maintain her social contacts in Y Distance and travel time as reasons for being always in a hurry: *“On Fridays getting from X to Z as early as possible”*, but *“this often doesn’t work because there is always something to be organized in X”* *“Departing from Z as late as possible”*	Since 2017, X no longer plays a role in her life
X → Y Y → X	Road distance (in km): 195 Travel time (by car): 2 h 3 min No further information provided by the interviewee		
Y → Z Z → Y	Road distance (in km): 92 Travel time (by car): 1 h 20 min		Since 2017, only 1 h travel time per direction, shorter visit intervals, and greater flexibility
Z → hospital hospital → Z	Road distance (in km): 57 Travel time (by car): 43		

Note: Distances and efforts for overcoming distances. Road distances and travel times are calculated using GoogleMaps.
